# Influence of aortic valve replacement on left ventricular mass and function in patients with aortic stenosis

**DOI:** 10.1186/1749-8090-8-S1-O19

**Published:** 2013-09-11

**Authors:** H Faisal, A Shaaban, M Ameen, H Eldomiaty, M Elkasas

**Affiliations:** 1 Cardiothoracic Surgery, Suez Canal University Hospital, Ismailia, Egypt

## Background

Aortic valve stenosis is associated with eccentric or concentric left ventricular (LV) hypertrophy and changes in the LV mass. The aim of this study was to evaluate the effect of AVR on LV mass and function, in patients with isolated aortic valve stenosis.

## Methods

34 patients with isolated aortic valve stenosis were studied using transthoracic echocardiography to assess LV mass and function before AVR and compared with postoperative changes occurring 6 months later in the LV dimensions and function.

## Results

Out of 34 patients, 20 (58.8%) were males and 14 (41.2%) were females. Mean age of the patients was 37.82±15.8 years. The mean left ventricular end diastolic dimension (LVED/BSA) was reduced by 2.11 from pre to post-operative (32.52 to 30.41, respectively; p<0.0001). The mean LVMI was reduced by 31.86 gm from pre to post-operative (174.15 to 142.3 gm, respectively; p<0.0001). the mean decrease of left ventricular mass index was 38.2±5.4 gm/m2 in patients received valve size 25; was 33.833±12.1 gm/m2 in patients received valve size 23; was 29.875±8.425 gm/m2 in patients received valve size 21, and was 23±19.621 gm/m2, in patients received valve size 19. The mean trans-aortic peak gradient was reduced by 41.36 from pre to post-operative (72.12 to 30.76, respectively; p<0.0001). The mean decrease of the transaortic peak gradient was 48.8±5.263 mmHg in patients received valve size 25 versus 33.286±17.414 mmHg in patients received valve size 19. The mean ejection fraction (%) was increased by 6.45 from pre to post-operative (54.26 to 60.71, respectively; p<0.0001).

## Conclusions

There was significant LV mass regression after aortic valve replacement in patients with pre existing aortic valve stenosis and significant improvement in LV function. The magnitude of reduction in left ventricular mass index is increasing steadily with the increase in the size of used prosthetic valve with considering the rules to avoid patient prosthesis mismatch.

